# Characterization of Five Podoviridae Phages Infecting *Citrobacter freundii*

**DOI:** 10.3389/fmicb.2016.01023

**Published:** 2016-06-29

**Authors:** Sana Hamdi, Geneviève M. Rousseau, Simon J. Labrie, Rim S. Kourda, Denise M. Tremblay, Sylvain Moineau, Karim B. Slama

**Affiliations:** ^1^Laboratoire des Microorganismes et Biomolécules Actives, Faculté des Sciences de Tunis, Université de Tunis-El ManarTunis, Tunisie; ^2^Département de Biotechnologie, Institut Supérieur des Sciences Biologiques Appliquées de Tunis, Université de Tunis El-ManarTunis, Tunisie; ^3^Département de Biochimie, de Microbiologie, et de Bioinformatique and PROTEO, Faculté des Sciences et de Génie, Félix d'Hérelle Reference Center for Bacterial Viruses, and GREB, Faculté de Médecine Dentaire, Université LavalQuébec City, QC, Canada

**Keywords:** *Citrobacter freundii*, pathogen, phages, virulent, *T7virus*, therapeutic applications

## Abstract

*Citrobacter freundii* causes opportunistic infections in humans and animals, which are becoming difficult to treat due to increased antibiotic resistance. The aim of this study was to explore phages as potential antimicrobial agents against this opportunistic pathogen. We isolated and characterized five new virulent phages, SH1, SH2, SH3, SH4, and SH5 from sewage samples in Tunisia. Morphological and genomic analyses revealed that the five *C. freundii* phages belong to the *Caudovirales* order, *Podoviridae* family, and *Autographivirinae* subfamily. Their linear double-stranded DNA genomes range from 39,158 to 39,832 bp and are terminally redundant with direct repeats between 183 and 242 bp. The five genomes share the same organization as coliphage T7. Based on genomic comparisons and on the phylogeny of the DNA polymerases, we assigned the five phages to the *T7virus* genus but separated them into two different groups. Phages SH1 and SH2 are very similar to previously characterized phages phiYeO3-12 and phiSG-JL2, infecting, respectively, *Yersinia enterocolitica* and *Salmonella enterica*, as well as sharing more than 80% identity with most genes of coliphage T7. Phages SH3, SH4, and SH5 are very similar to phages K1F and Dev2, infecting, respectively, *Escherichia coli* and *Cronobacter turicensis*. Several structural proteins of phages SH1, SH3, and SH4 were detected by mass spectrometry. The five phages were also stable from pH 5 to 10. No genes coding for known virulence factors or integrases were found, suggesting that the five isolated phages could be good candidates for therapeutic applications to prevent or treat *C. freundii* infections. In addition, this study increases our knowledge about the evolutionary relationships within the *T7virus* genus.

## Introduction

Members of the Gram-negative *Enterobacteriaceae* have caused significant diseases throughout human history. They are responsible for many human infections in the intestine, urinary tract, bloodstream, and wounds (Abbott, 2011; Shanks et al., 2012). The genus *Citrobacter* belongs to this bacterial family, although it was originally classified within the genus *Salmonella* due to biochemical and serological similarities (Harhoff, 1949; Ewing and Davis, 1972). *Citrobacter freundii* is the type species of this genus, with a genome size of ~5 Mb and a G+C content of 50 to 52% (Kumar et al., 2013; Kimura et al., 2014). *C. freundii* is commonly found in soil, water, foods, and the intestinal tracts of animals and humans (Drelichman and Band, 1985). Some strains of *C. freundii* can also cause opportunistic infections in humans and animals, which are becoming more difficult to treat due to increased antibiotic resistance. As such, *C. freundii* infections have become a public health concern (Samonis et al., 2008; Antonelli et al., 2015; Campos et al., 2015) and alternatives or adjuncts to antibiotic treatment are required.

In this context, lytic/virulent phages are being re-investigated as potential antimicrobial agents to either combat bacterial diseases or to stop the dissemination of multi-resistant bacteria. The potential of phages to control or treat bacterial diseases has been previously demonstrated (Smith and Huggins, 1982; Slopek et al., 1983). However, their use was mostly abandoned for several well-documented reasons including the inability to purify phage preparations from bacterial components, the lack of understanding of basic phage biology, the inability to differentiate temperate from lytic phages, narrow host ranges, the development of phage-resistant bacterial mutants, and the inherent difficulties of patenting phages and their use. It is believed that progress has been made to overcome most, if not all, these difficulties (Carlton, 1999; Loc-Carrillo and Abedon, 2011).

Several phages infecting various strains of *C. freundii* have been recently characterized. Six of them belong to the *Myoviridae* family [double-stranded DNA genome (dsDNA), contractile tail] and were isolated from water samples in Texas. Their genomic characterization indicated that three of these phages (Moon, Miller, Merlin) are related to the *T4virus* genus (Edwards et al., 2015; Hwang et al., 2015; LeSage et al., 2015) while the other three (Mordin, Michonne, Moogle) are related to the *Felixo1virus* genus (Bernal et al., 2015; Guan et al., 2015; Nguyen et al., 2015). The complete genomic sequence of the *C. freundii* phage Stevie is also available (Shaw et al., 2015). This *Siphoviridae* phage (dsDNA, noncontractile tail), which was isolated from a dirt sample in Texas, is related to the *T1virus* genus. Phages of the *Podoviridae* family (dsDNA, short tail) can also infect *C. freundii* strains as the podophage LK1 was isolated from sewage and its genome size was estimated to be 20–23 kb (Chaudhry et al., 2014). The podophage phiCFP-1 was isolated from sewage in China and classified as a *T7virus* with a genome of 38,625 bp with 43 *orfs* and direct terminal repeats of 229 bp (Zhao et al., 2015).

Phages belonging to the *T7virus* genus are particularly interesting for therapeutic applications as they are usually easy to culture and have a short lytic cycle. They also have smaller genomes and a conserved organization, which facilitates their in-depth analysis. Their genomes can be divided into three transcriptional regions including early-, middle-, and late-expressed genes (Scholl and Merril, 2005; Zhu et al., 2010). As for the prototype coliphage T7, the genes of these phages can be transcribed due to an efficient phage-encoded RNA polymerase that specifically recognizes a set of conserved promoters dispersed throughout the phage genome (Chen and Schneider, 2005; Huang et al., 2012).

Here, we describe five lytic *Podoviridae* phages infecting *C. freundii* isolated from sewage samples in Tunisia. Their analyses showed that they belong to the *Autographivirinae* subfamily and they share similarities with phages infecting other *Enterobacteriaceae.*

## Materials and methods

### Bacterial strains, phage isolation, and culture conditions

Five bacterial isolates were obtained by plating Tunisian wastewater samples on *Salmonella*-*Shigella* agar (Biokar) and incubating the plates for 24 h at 37°C. The species of each bacterial isolate was determined by 16S rRNA sequencing and API 20 E strip (BioMérieux). *C. freundii* strains were genotyped using multi-locus sequence typing (MLST) of seven housekeeping genes (*aspC, clpX, fadD, mdh, arcA, dnaG*, and *lysP*) as described previously (Bai et al., 2012). The allelic profile and sequence type (ST) of each strain was identified using the MLST database website (http://pubmlst.org/cfreundii/). Evolutionary analyses were conducted with MEGA7 (Kumar et al., 2016). The neighbor-joining phylogenetic tree (Saitou and Nei, 1987) of the five strains was generated from the concatenated sequences of the seven loci. The evolutionary distances were computed using the Maximum Composite Likelihood method (Tamura et al., 2004) and are in the units of the number of base substitutions per site.

Two *C. freundii* isolates were used as hosts for phage isolation. Water samples were obtained from four different areas in Tunis (Table 1). One millilitre of the filtered water samples was mixed with 1 ml of an overnight bacterial culture in 3 ml of Brain Heart Infusion broth (BHI) (Biokar or BD). After incubation for 24 h at 37°C, the mixtures were centrifuged and 4 μl of each filtered-supernatant was spotted on a fresh bacterial lawn. After incubation at 37°C for 24 h, phage lysis zones were picked with a sterile truncated tip and amplified in the presence of their respective host in BHI for 24 h at 37°C. Then, the mixtures were centrifuged and the supernatants filtered. Isolated plaques were obtained using the double-layer agar method and picked with a sterile truncated tip. This step was repeated three times to ensure phage purity. Phages and bacterial strains were deposited at the Félix d'Hérelle Reference Center for Bacterial Viruses of the Université Laval (www.phage.ulaval.ca) under the following names: phages SH1 (HER 516), SH2 (HER 517), SH3 (HER 518), SH4 (HER 519), and SH5 (HER 520) as well as *C. freundii* strains CF3 (HER 1518) and CF5 (HER 1516).

**Table 1:** **Origins of phages and their host strains**.

**Phage**	**Origins of phages**	**Host strain**	**Origins of strain**
SH1	Wadi of Khaznadar	CF5	Entry water treatment plant of Menzah1
SH2	Wastewater from Mellassine	CF5	
SH3	Office of national sanitation of Ksar Said	CF3	Entry water treatment plant of Gammarth
SH4	Wastewater from Mellassine	CF3	
SH5	Wadi of Ezzouhour city	CF3	

### Microbiological assays

The host range of the five phages was determined by spotting 4 μl of various serial dilutions (10^0^ to 10^−7^) of a phage lysates on BHI soft agar (0.75% agar) containing one bacterial strain. After overnight incubation at 37°C, plates were examined for the presence of isolated plaques in the spotted areas, which indicated a full phage lytic cycle on the host. The host range was tested on 5 *C. freundii* (this study), one *Cronobacter turicensis* (290708/07) and 25 bacterial strains available at the Félix d'Hérelle Reference Center for Bacterial Viruses of the Université Laval: 10 *Escherichia coli* (HER1024, HER1040, HER1144, HER1255, HER1462, HER1155, HER1290, HER1022, HER1213, and HER1445), two *Shigella dysenteriae* (HER1020 and HER1031), one *Shigella sonnei* (HER1043), two *Salmonella* Paratyphi (HER1045, HER1220), one *Salmonella* Typhi (HER1038), two *Salmonella* Typhimurium (HER1023, HER1095), two *Salmonella* Newport (HER1185 and HER1019), one *Salmonella* Heidelberg (HER1428), one *Salmonella* Senftenberg (HER1397), and 3 *Yersinia enterocolitica* (HER1249, HER1071, HER1072). Phage susceptibility to pH (2 to 10) was also determined in BHI broth with the pH adjusted using hydrochloric acid (HCl) or sodium hydroxide (NaOH). One hundred microlitre of each phage lysate at >10^9^PFU/ml were mixed with 900 μl of media for each pH condition and incubated at 37°C for 60 min. Phage titer was then determined using the double-layer agar method.

### Electron microscopy

Phages were prepared and observed as described previously (Fortier and Moineau, 2007). The reported dimensions are the means of at least ten virions stained with uranyl acetate (2%).

### Phage structural proteins

Phages were precipitated from lysates (1L) with 10% polyethylene glycol (PEG) 8000 and 2922g of sodium chloride then concentrated using a discontinuous CsCl gradient followed by a continuous CsCl gradient, as described previously (Chibani Azaïez et al., 1998; Sambrook and Russel, 2001). A purified phage sample was sent directly for structural protein identification by liquid chromatography/tandem mass spectrometry (LC-MS/MS) at the Plateforme Protéomique, Centre de Génomique de Québec (Université Laval). A custom database was generated using the putative predicted proteins. Results were analyzed using Scaffold Proteome software version 4.4.5.

### Genome sequencing and bioinformatics analyses

Phage DNA was extracted from high titer phage lysates using a Plasmid Maxi Kit (Qiagen) with modifications described elsewhere (Deveau et al., 2002). Phage DNA was prepared for sequencing using the Nextera XT DNA library preparation kit (Illumina) according to the manufacturer's instructions. The libraries were then sequenced on a MiSeq system using a MiSeq reagent kit v2 (Illumina, 500 cycles). *De novo* assembly was performed with Ray assembler version 2.2.0 using *k*-mer sizes of 21, 51, 96, 31, and 51 and we obtained mean coverage depths for each single phage contig of 2717, 1643, 3804, 134, and 2431 for SH1, SH2, SH3, SH4, and SH5, respectively. Coverage was calculated with Samtools. Open reading frames (ORFs) were identified using ORF Finder (Rombel et al., 2002) and GeneMark (Lukashin and Borodovsky, 1998) then confirmed by visual inspection for the presence of a Shine-Dalgarno sequence close to a start codon (AUG, UUG or GUG) using BioEdit 7.2.0 (Hall, 1999). ORFs were considered if they contained at least 30 amino acids (aa). Similarities with known proteins were searched with BLAST. Hits were considered when the *E*-value was lower than 10^−3^. The percentage of identity between proteins was calculated by dividing the number of identical residues by the size of the smallest protein. The theoretical molecular weight (MW) and isoelectric point (pI) of the ORFs were calculated using the Compute pI/MW tool (http://web.expasy.org/compute_pi/).

### Determination of genome ends

To confirm the direct terminal repeats, primers adjacent to the predicted terminal ends were designed using Primer-BLAST at NCBI. The putative ends were established by aligning the genome termini with similar phage genomes using ClustalW2 (http://www.ebi.ac.uk/Tools/msa/clustalw2/). The primers were used to sequence directly from the phage DNA at the sequencing and genotyping platform of the Université Laval using the ABI data 3730XL DNA analyzer. The primers used are described in Table 2. Terminal repeat sequences were determined using Staden software (version 1.7.0) (Staden, 1996).

**Table 2:** **Primers used to determine the terminal repeats**.

**Phage**	**Forward primer (5′-3′)**	**Reverse primer (5′-3′)**
SH1	GCCTCACTGTTCCGTCATTT	CAACTGAAAGGAGGTGGCTC
SH2	TGTCTCAGGGAGTGGCTTTA	GCTCAATGTTACGCTTGCTG
SH3	GCCCTACCCCAGTCTATCAT	CTATCCCTACGCCATCTTGC
SH4/SH5	CTGCTGTTCTACTTGCTGCT	GCTATGGTCCCTGACTGCTA

### DNA polymerase phylogeny

The DNA polymerase sequence dataset used for phylogeny included phage proteins from different families and genera (Labrie et al., 2013). The sequences were aligned using MAFFT with the E-INS-i parameter (Katoh and Standley, 2013). The alignment was then processed to generate the tree as previously described (Mercanti et al., 2015). Briefly, the best amino-acid substitution model implemented in PhyML 3.0 to calculate the best tree was predicted with ProtTest 3.2 (Darriba et al., 2011). The Shimodaira-Hasegawa-like procedure was used to determine the branch support values (Shimodaira, 2002). Finally, Newick utility package (Junier and Zdobnov, 2010) and ITOL (Letunic and Bork, 2011) were used to render the tree.

### Nucleotide sequence accession numbers

The annotated phage genomic sequences were deposited in GenBank under the numbers KU687347 (SH1), KU687348 (SH2), KU687349 (SH3), KU687350 (SH4), KU687351 (SH5).

## Results

### Isolation of bacteria and phages

**Figure 1:**
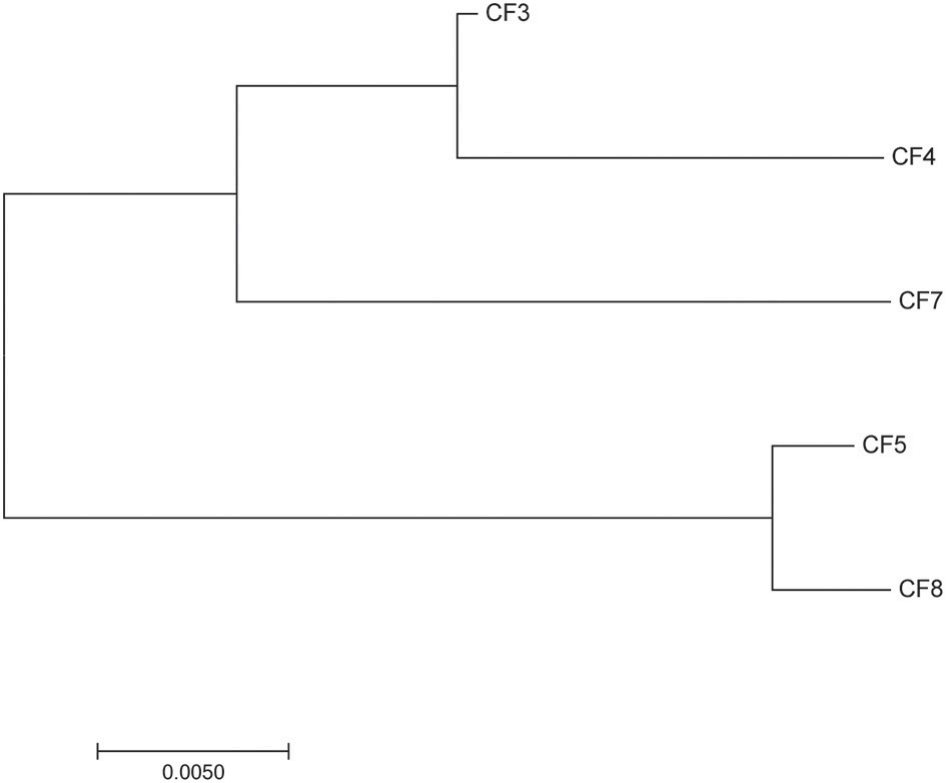
**Neighbor-joining phylogenetic tree of the five strains of *Citrobacter freundii***.

Five bacterial strains were isolated from different wastewater samples. Gram staining showed Gram negative bacilli. Sequencing of 16S rRNA and API 20E strip identification revealed that they belong to the *C. freundii* species. MLST analyses showed that the five strains also belong to different genotypes, CF5 belong to ST19 and the four other strains belong to four novel and different ST. Phylogenetic analyses (Figure 1) revealed that CF3, CF4, and CF7 belonged to a different branch from CF5 and CF8. Two *C. freundii* isolates (CF3 and CF5) were selected from each branch and used as host organisms to isolate phages.

**Figure 2:**
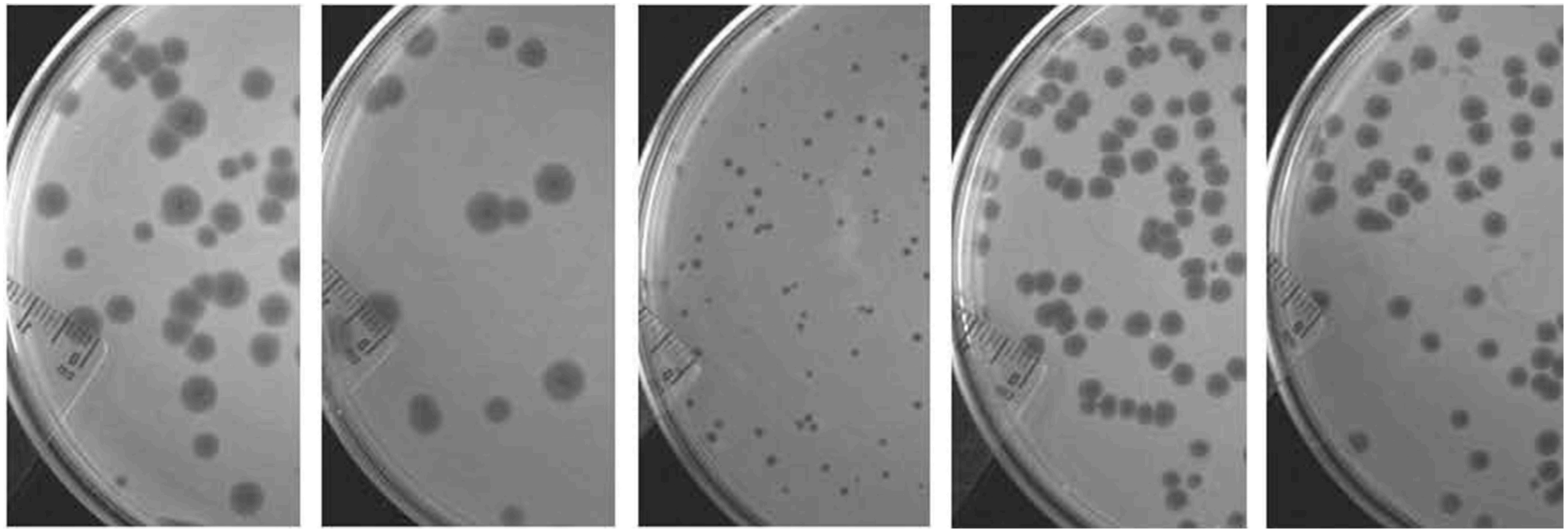
**Plaques formed by phages SH1, SH2, SH3, SH4, and SH5, respectively, from left to right on their host strains of *C. freundii* after an overnight incubation at 37°C**.

A total of five virulent phages, SH1, SH2, SH3, SH4, and SH5, were isolated from four sewage samples (Table 1). For phages SH1 and SH2, plaques of 2 mm in diameter appeared after only 3 h of incubation at 37°C and the plaques became larger with diameters ranging from 4 to 6 mm after overnight incubation, as shown in Figure 2. Phage SH3 produced smaller plaques of 1 mm in diameter while phages SH4 and SH5 produced plaques of about 3 mm in diameter.

The host range of the five phages was determined using the 31 Gram-negative bacterial strains described in the Materials and Methods section. Phages SH1 and SH2 were able to lyse their host strain, *C. freundii* CF5, and *S.* Typhi HER1038. Phage SH3 was able to lyse its host strain, *C. freundii* CF3 and *C. freundii* CF4. Phages SH4 and SH5 lysed their host strain, *C. freundii* CF3, as well as *C. freundii* CF4 and *C. turicensis* 290708/7.

### Sensitivity to pH

The five phages were tested for their susceptibility to different pH conditions. They were exposed to pHs ranging from 2 to 10 for 1 h at 37°C. All phages were completely inactivated when exposed to pH 2 and pH 3. A 10-fold reduction in phage titer was also noticed at pH 4. All phage suspensions were stable from pH 5 to pH 10.

### Morphological characteristics

**Figure 3:**
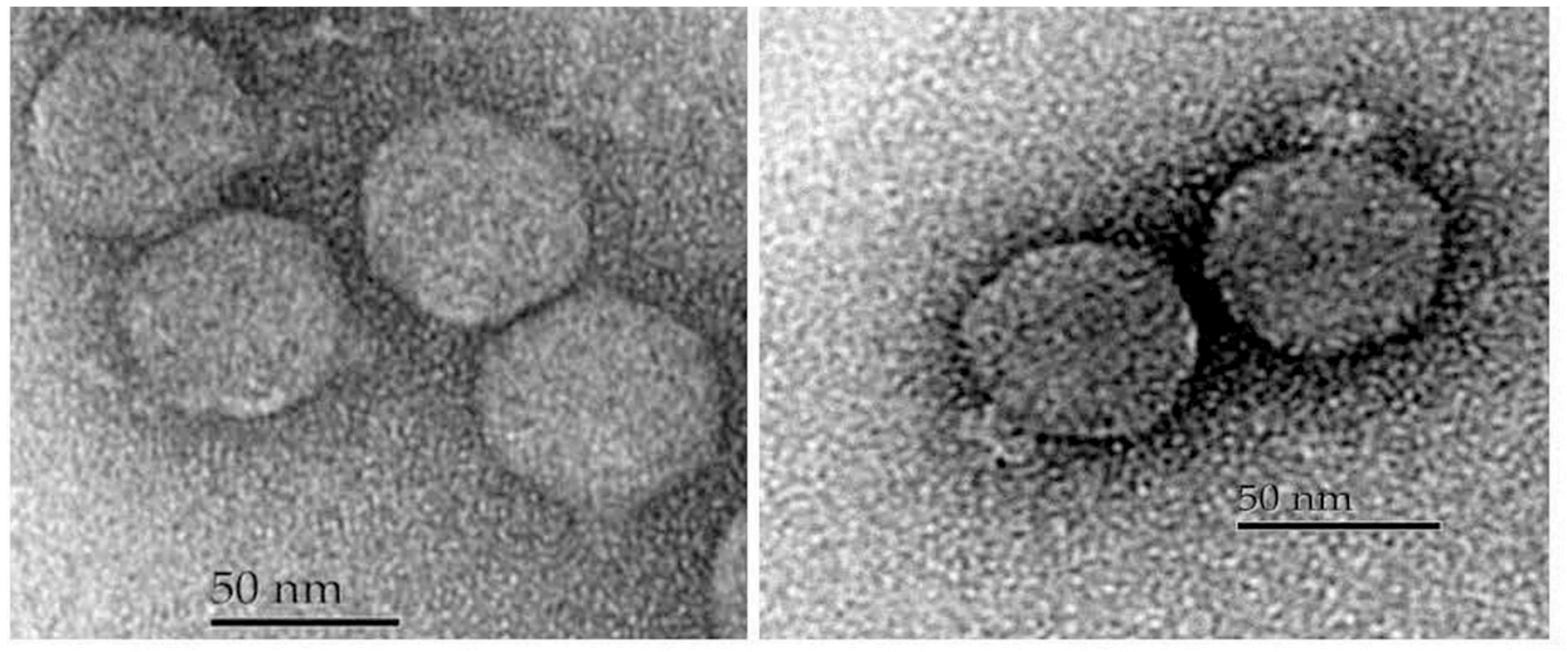
**Electron micrographs of phages SH1 (left) and SH3 (right)**.

Negatively stained purified phages were observed with an electron microscope and all five possessed an icosahedral capsid and small non-contractile tail (Figure 3, Table 3). However, the tips of the tails differed which led us to divide them into two morphological groups. The first group included phages SH1 and SH2, which had a narrower base plate compared to the second group, which included phages SH3, SH4, and SH5 (Figure 3). Nonetheless, their overall morphology allowed us to classify the five phages into the *Caudovirales* order and the *Podoviridae* family.

**Table 3:** **Morphological and genomic characteristics of the five isolated phages and phage T7**.

**Phage**	**Capsid (nm)**	**Tail (nm)**		**Genome size (bp)**	**GC%**	**Terminal repeat (bp)**
		**Width**	**Length**			
**SH1**	61 ± 1.6	14 ± 0.9	12 ± 1.0	39,434	51.0	230
**SH2**	58 ± 2.5	14 ± 1.4	10 ± 1.1	39,158	50.7	242
**SH3**	65 ± 1.4	21 ± 1.1	13 ± 1.0	39,444	50.6	183
**SH4**	67 ± 1.3	29 ± 2.4	16 ± 1.2	39,274	52.6	190
**SH5**	65 ± 1.1	27 ± 1.8	16 ± 2.0	39,832	52.5	190
T7^*^	56	14	9	39,936	50	160

* According to (Ackermann and Nguyen, 1983; Dunn et al., 1983).

### Genomic characteristics

**Figure 4:**
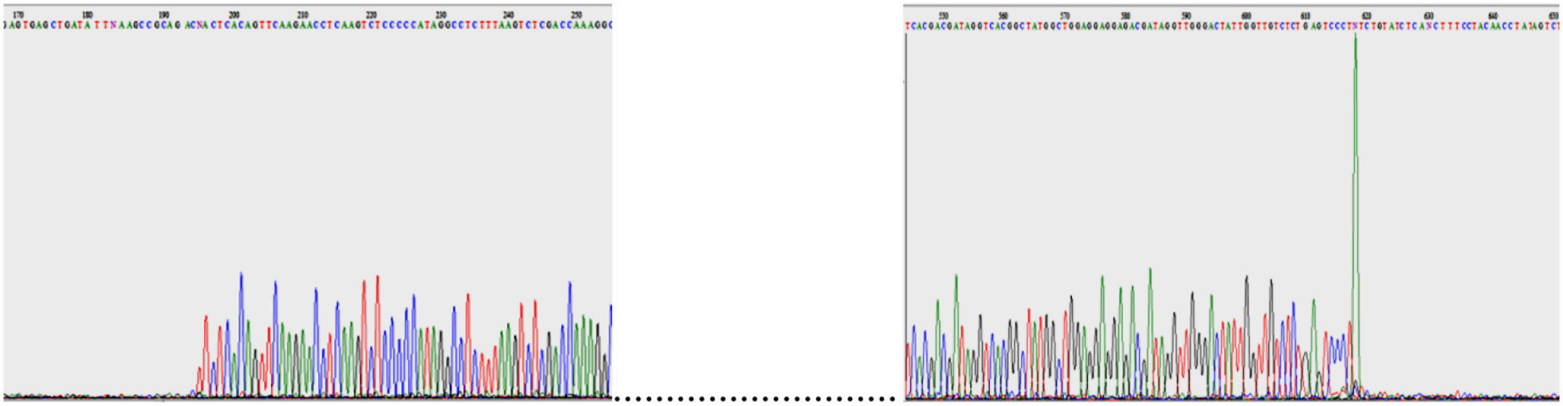
**SH5 genome sequencing with both reverse and forward primers**.

The double-stranded DNA of the five phages was extracted and sequenced. The genome size of these phages ranged from 39,158 to 39,832 bp, which was similar to that of coliphage T7 (39,936 bp) (Table 3). The GC contents of the phage genomes were similar to that of their *C. freundii* hosts, 50 to 51% (Frederiksen, 2015). After genome alignments with similar phages, primers adjacent to the predicted terminal ends were used to directly sequence the phage genomic DNA. As expected, the sequencing signal dropped at the end of the genome (Figure 4) and this was used to determine the position of the terminal ends and their sequences. The last adenine at the end of the repeated sequences was not considered because it is added by the polymerase (Clark, 1988; Garneau et al., 2010). Our analyses revealed that the five *Podoviridae* phage (podophage) genomes contained direct terminal repeats at both ends (Table 3). The length of the direct terminal repeats of phages SH1 (230 bp) and SH2 (242 bp) were similar to that of *Yersinia* phage phiYeO3-12 (232 bp; Pajunen et al., 2001), *Salmonella* phage phiSG-JL2 (230 bp; Kwon et al., 2008), and *Citrobacter* phage phiCFP-1 (229 bp; Zhao et al., 2015). Terminal repeat lengths of SH3 (183 bp), SH4 (190 bp), and SH5 (190 bp) were close to the length of coliphage K1F (179 bp; Scholl and Merril, 2005).

### Genome organization

**Figure 5:**
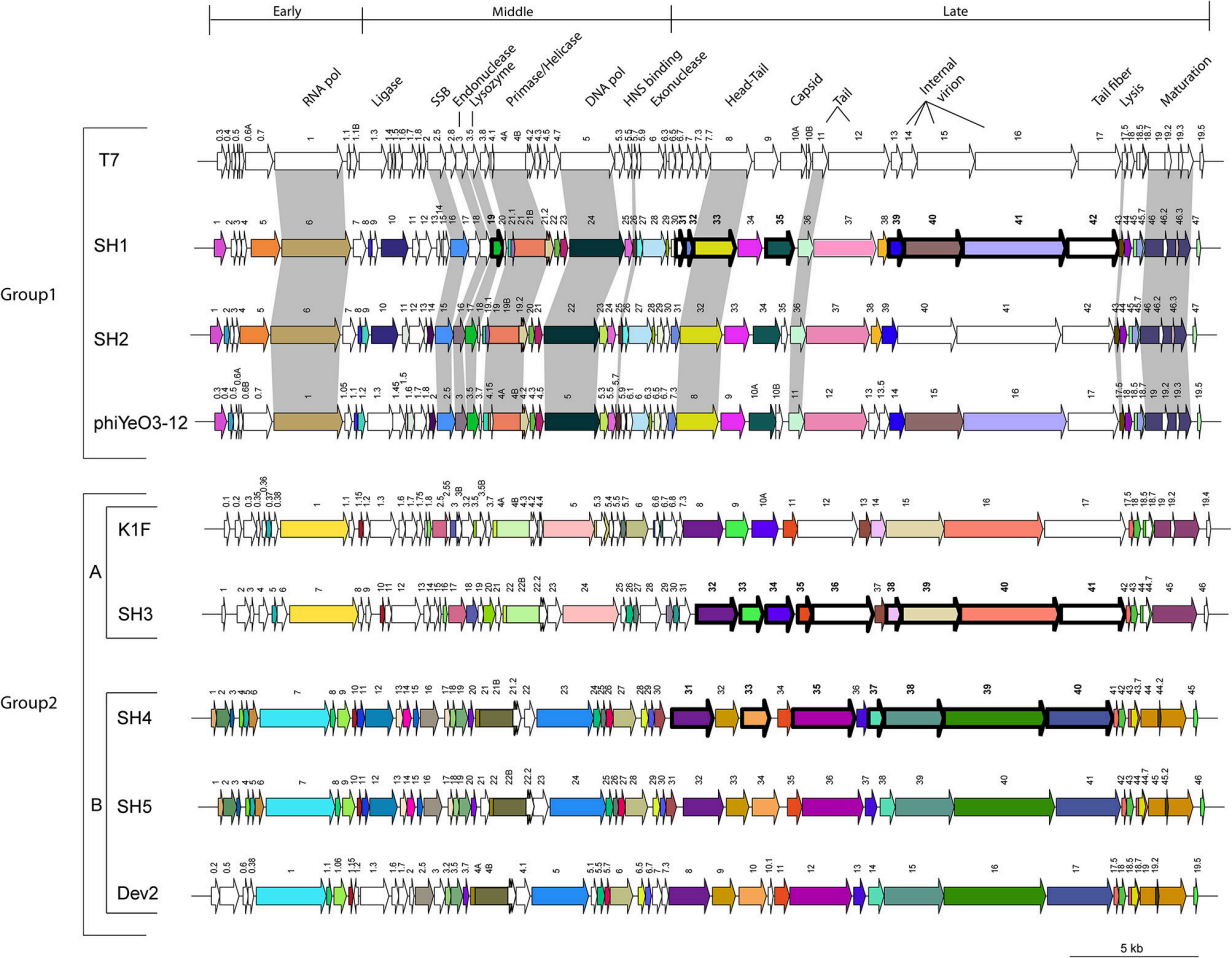
**Schematic representation of the genomic organization of phage T7 (NC_001604.1) compared to phages SH1, SH2, phiYeO3-12 (NC_001271.1), K1F (NC_007456.1), SH3, SH4, SH5, and Dev2 (NC_023558.1)**. Each arrow represents an ORF. Deduced ORFs sharing 95% amino acid identity are represented with the same color. Gray shading indicates ORFs whose translated products share 80% identity with the ones of phage T7. Finally, arrows with thick outlines and bold numbers represent structural proteins detected by LC-MS/MS.

Analyses of the predicted *orfs* in the genomes of the five newly isolated podophages revealed that they all have the same transcriptional orientation and use only ATG as an initiation codon (Tables 4, 5). Comparative genome analyses also indicated that these phages were affiliated with the *Autographivirinae* subfamily and the *T7virus* genus. Similar to the morphological groupings, we could also divide the five phage genomes into subgroups (Figure 5). The first group included phages SH1 and SH2, which had high identity (80%) to genes of *Yersinia* phage phiYeO3-12 as well as coliphages T7 and T3. The second phage group (SH3, SH4, and SH5) could be divided into two subgroups. Group 2A included phage SH3, which was close to coliphage K1F, while group 2B was comprised of phages SH4 and SH5, which are similar to *Cronobacter* phage Dev2.

**Table 4:** **Features of the ORFs of phage SH2, identity with SH1, predicted functions of proteins, and best matches with database**.

				**Predicted protein**						**Aligned protein**		
**ORF**	**SH1 ORF,%**	**Start (bp)**	**Stop (bp)**	**Size (aa)**	**MW(kDa)**	**pI**	**SD sequence TAAGGAGGT (5′-3′)^a^**	**Predicted function**	**BLAST (extent; %aa identity)^b^**	***E* Value**	**Size (aa)**	**Accession number**
1	ORF1, 99	948	1406	152	17.0	7.6	TGAGGTAACaccaa**ATG**	S-adenosyl-l-methionine hydrolase	gp0.3 [*Yersinia* phage phiYeO3-12] (152/152; 100%)	4.00E-110	152	NP_052065.1
2	ORF2, 87	1479	1679	66	7.5	6.8	ATAGGACTAacacc**ATG**		gp0.45 [*Salmonella* phage phiSG-JL2] (65/66; 98%)	4.00E-41	66	YP_001949746.1
3	ORF3, 74	1699	1857	52	6.0	9.7	ACAGGAGGAttagca**ATG**		hypothetical protein [*Enterobacter* phage E-4] (49/52; 94%)	3.00E-26	52	AKA61646.1
4	ORF4, 94	1854	2051	65	7.8	10.5	TGGTGAAACacgc**ATG**		hypothetical protein [*Enterobacter* phage E-4] (63/65; 97%)	2.00E-38	65	AKA61645.1
5	ORF5, 95	2073	3182	369	42.3	7.1	TAAGGACACactgaa**ATG**	Protein kinase	gp0.7 [*Yersinia* phage phiYeO3-12] (346/369; 94%)	0.0	369	NP_052070.1
6	ORF6,99	3253	5907	884	98.8	7.1	CAATGAGGTaagca**ATG**	RNA polymerase	DNA-directed RNA polymerase [*Enterobacter* phage E-2] (881/884; 99%)	0.0	884	AKA61565.1
7	ORF7, 90	6008	6502	164	19.6	9.2	TAAGAGGATtacttt**ATG**		gp1.05 [*Salmonella* phage phiSG-JL2] (76/165; 46%)	3.00E-38	169	YP_001949751.1
8	ORF8, 100	6593	6733	46	5.9	10.9	TAAGATACT**ATG**		gp1.1 [*Yersinia* phage phiYeO3-12] (46/46; 100%)	2.00E-23	46	NP_052073.1
9	ORF9, 69	6736	7008	91	10.3	7.9	AGTGGAACTaatg**ATG**	Deoxyguanosine triphospho-hydrolase inhibitor	gp1.2 [*Salmonella* phage phiSG-JL2] (90/91; 99%)	1.00E-160	92	YP_001949753.1
10	ORF10, 98	7103	8119	338	38.4	5.0	TGAGGAACAaccgt**ATG**	DNA ligase	gp1.3 [*Salmonella* phage phiSG-JL2] (333/338; 99%)	0.0	338	YP_001949754.1
11	ORF11, 94	8291	8548	85	9.9	11.2	TAAGGAGACaacatc**ATG**	LysR family transcriptional regulator	gp1.6 [*Yersinia* phage phiYeO3-12] (85/85; 100%)	2.00E-53	85	NP_052078.1
12	ORF12, 66	8548	9132	194	21.6	9.1	TAAGGAGGTgctgta**ATG**	Nucleotide kinase	phiYe-F10_00014 [*Yersinia* phage phiYe-F10] (163/190; 86%)	6.00E-115	190	AKQ06773.1
13	ORF14, 85	9119	9256	45	5.3	5.2	TAAGGGGCTgtgct**ATG**		AVU28_gp19 [*Enterobacter* phage E-3] (42/45; 93%)	1.00E-21	45	AKA61598.1
14	ORF15, 62	9253	9489	78	8.8	4.8	TAAGGAGGCcaataa**ATG**	Bacterial RNA polymerase inhibitor	ORF13 [*Yersinia* phage vB_YenP_AP5] (77/78; 99%)	1.00E-50	78	AIM40358.1
15	ORF16, 98	9542	10240	232	26.0	4.8	AAAGGAGAAacatc**ATG**	Single-stranded DNA-binding	phiYe-F10_00017 [*Yersinia* phage phiYe-F10] (227/232; 98%)	4.00E-166	232	AKQ06776.1
16	ORF18, 73	10240	10701	153	17.6	9.5	CGAGGACTTcta**ATG**	Endonuclease	gp3 [*Yersinia* phage phiYeO3-12] (152/153; 99%)	2.00E-105	153	NP_052083.1
17	ORF19, 99	10694	11149	151	16.9	9.0	TAAAGAAAA**ATG**	N-acetylmuramoyl-l-alanine amidase	AVU28_gp15 [*Enterobacter* phage E-3] (151/151; 100%)	2.00E-108	151	AKA61594.1
18	ORF20, 100	11154	11261	35	4.2	8.5	GAGGGTGATacc**ATG**		3.7 protein [*Yersinia* phage phiYeO3-12] (35/35; 100%)	2.00E-15	35	NP_052086.1
19	ORF21,99	11328	13028	566	6.3	5.2	TAAGGAATGtaca**ATG**	Primase/Helicase	gp4A [*Salmonella* phage phiSG-JL2] (560/566; 99%)	0.0	566	YP_001949764.1
19.1	ORF21.1, 94	11362	11517	51	5.6	6.5	TCTTTCTGTttc**ATG**		hypothetical protein [*Enterobacteria* phage T3] (49/51; 96%)	1.00E-26	51	AGM10719.1
19B	ORF21B,99	11514	13028	504	55.9	5.1	GGAGGCAGTaaccct**ATG**	Primase/Helicase	Primase/Helicase protein [*Enterobacter* phage E-3] (501/504; 99%)	0.0	504	AKA61593.1
19.2	ORF21.2, 97	12748	13077	109	12.1	6.7	GAAGGGAAAaccac**ATG**		gp4.2 [*Enterobacteria* phage T3] (103/109; 94%)	2.00E-66	109	NP_523317.1
20	ORF22, 97	13124	13336	70	7.7	10.0	ATAGGAGACacatc**ATG**		gp4.3 [*Yersinia* phage phiYeO3-12] (68/70; 97%)	4.00E-29	70	NP_052091.1
21	ORF23, 100	13349	13633	94	10.7	9.9	TAAGGAGCGaacact**ATG**		gp4.5 [*Yersinia* phage phiYeO3-12] (94/94; 100%)	2.00E-62	94	NP_052092.1
22	ORF24, 98	13701	15815	704	79.8	6.5	AAAGGAGGGcatt**ATG**	DNA polymerase	gp5 [*Yersinia* phage phiYeO3-12] (700/704; 99%)	0.0	704	NP_052093.1
23		15825	16157	110	13.0	9.5	TAAGGAGGAttt**ATG**	Homing Endonuclease	gp5.3 [*Yersinia* phage phiYeO3-12] (110/110; 100%)	2.00E-75	110	NP_052095.1
24	ORF25, 99	16135	16437	101	11.1	6.3	AAAGGAGAAacatt**ATG**	HNS binding	gp5.5 [*Yersinia* phage phiYeO3-12] (100/101; 99%)	4.00E-66	101	NP_052097.1
25	ORF26, 100	16553	16762	69	7.3	9.8	TTGGGAGGTactcta**ATG**		gp5.7 [*Yersinia* phage phiYeO3-12] (69/69; 100%)	8.00E-42	69	NP_052098.1
26	ORF27, 99	16705	16941	60	8.8	4.2	CAATGGTGGagc**ATG**		gp5.9 [*Yersinia* phage phiYeO3-12] (60/60; 100%)	1.00E-34	60	NP_072071.1
27	ORF28, 99	16938	17849	303	34.7	4.9	GGAGGATGAcga**ATG**	Exonuclease	AVU28_gp07 [*Enterobacter* phage E-3] (301/303; 99%)	0.0	303	AKA61586.1
28	ORF29, 100	17831	17944	37	4.1	9.7	CAAGGAGATttactt**ATG**		gp6.3 [*Yersinia* phage phiYeO3-12] (37/37; 100%)	1.00E-15	37	NP_052102.1
29	ORF30, 96	18039	18284	81	9.3	5.9	TTAAGAGGTgaattt**ATG**		gp6.5 [*Yersinia* phage phiYeO3-12] (79/81; 98%)	4.00E-51	81	NP_052103.1
30	ORF31, 69	18289	18540	83	8.8	9.1	ACAGGAGTAattat**ATG**	Head	gp6.7 [*Yersinia* phage vB_YenP_AP5] (83/83; 100%)	2.00E-49	83	YP_009102822.1
31	ORF32, 96	18568	18888	106	11.0	9.8	TAGGGAGAAacatc**ATG**	Host specificity protein B	gp7.3 [*Salmonella* phage phiSG-JL2] (105/106; 99%)	1.00E-62	106	YP_001949779.1
32	ORF33, 99	18899	20506	535	58.6	4.5	TAAGGAGGActga**ATG**	Head-to-tail joining	gp8 [*Yersinia* phage phiYeO3-12] (535/535; 100%)	0.0	535	NP_052106.1
33	ORF34, 97	20608	21540	310	33.8	4.3	TTAGGAGATttaaca**ATG**	Capsid assembly	ORF30 [*Citrobacter* phage phiCFP-1] (303/310; 98%)	0.0	310	AKA62148.1
34	ORF35, 95	21697	22740	347	36.8	6.2	TAAGGAGATtcaac**ATG**	Minor and Major capsid	10A [*Yersinia* phage phiYe-F10] (344/346; 99%)	0.0	347	AKQ06793.1
35		22779	23012	77	7.4	4.5	TCAGAAGACt**ATG**	Minor capsid	AVU28_gp35 [*Enterobacter* phage E-3] (73/77; 95%)	1.00E-39	77	AKA61614.1
36	ORF36, 99	23125	23715	196	22.2	4.5	ACAGGAGGTaacatc**ATG**	Tail tubular A	gp11 [*Yersinia* phage phiYeO3-12] (196/196; 100%)	1.00E-141	196	NP_052110.1
37	ORF37, 99	23731	26136	801	89.8	5.9	CAAGGAGGCtct**ATG**	Tail tubular B	gp12 [*Salmonella* phage phiSG-JL2] (797/801; 99%)	0.0	801	YP_001949785.1
38	ORF38, 98	26209	26619	136	15.8	5.6	TAAAGCATT**ATG**	Internal virion A	AXI78_gp37 [*Enterobacter* phage E-2] (134/136; 99%)	4.00E-95	136	AKA61575.1
39	ORF39, 100	26622	27215	197	21.2	9.4	GTAGGAGGTaact**ATG**	Internal virion B	gp14 [*Yersinia* phage phiYeO3-12] (194/197; 98%)	4.00E-136	197	NP_052114.1
40	ORF40, 80	27218	29461	747	84.6	6.1	CCGGGAGGTaata**ATG**	Internal virion C	ORF37 [*Citrobacter* phage phiCFP-1] (711/747; 95%)	0.0	747	AKA62155.1
41	ORF41, 83	29484	33452	1322	144.2	6.7	TAAGGAGGCtcc**ATG**	Internal virion D	ORF38 [*Citrobacter* phage phiCFP-1] (1296/1322; 98%)	0.0	1322	AKA62156.1
42	ORF42, 92	33524	35500	658	69.9	6.0	AAAGGAGGTcac**ATG**	Tail fiber	gp17 [*Salmonella* phage phiSG-JL2] (593/658; 90%)	0.0	658	YP_001949790.1
43	ORF43, 99	35511	35714	67	7.4	6.1	TAAGGAGGAcata**ATG**	Lysis	gp17.5 [*Yersinia* phage phiYeO3-12] (66/67; 99%)	5.00E-39	67	NP_052118.1
44	ORF44, 100	35718	35984	88	9.9	4.7	CAAGGAGTAacct**ATG**	DNA packaging A	gp18 [*Salmonella* phage phiSG-JL2] (88/88; 100%)	1.00E-55	88	YP_001949792.1
45	ORF45, 99	36062	36526	150	17.3	9.2	ATGGGAGGTgtt**ATG**	Endopeptidase Rz	ORF42 [*Citrobacter* phage phiCFP-1] (152/154; 99%)	1.00E-107	154	AKA62160.1
45.7	ORF45.7, 99	36189	36443	84	9.3	9.8	TAATCCAAA**ATG**		gp18.7 [*Salmonella* phage phiSG-JL2] (83/84; 99%)	9.00E-52	84	YP_001949794.1
46	ORF46, 99	36501	38264	587	66.6	5.3	TAAGGAGATgcaga**ATG**	DNA packaging B	gp19 [*Salmonella* phage phiSG-JL2] (581/587; 99%)	0.0	587	YP_001949795.1
46.2	ORF46.2, 93	37213	37383	56	6.1	10.0	GAAGACTTGtact**ATG**		19.2 protein [*Yersinia* phage phiYeO3-12] (56/56; 100%)	2.00E-28	77	NP_052123.1
46.3	ORF46.3, 95	37687	37815	42	4.7	11.9	TGGCGGGTTccgcg**ATG**		19.3 protein [*Yersinia* phage phiYeO3-12] (42/42; 100%)	1.00E-19	42	NP_052124.1
47	ORF47, 96	38509	38658	49	5.5	7.9	AAAGGAGGTggctcA**ATG**		AVU28_gp23 [*Enterobacter* phage E-3] (48/49; 98%)	9.00E-25	49	AKA61602.1

a Start codon indicated in boldface; Match to SD sequence is indicated by underlining; SD position is indicated in uppercase.

b The number of identical amino acids/The total number of amino acids of smallest protein.

**Table 5:** **Features of the ORFs of phage SH5, identity with SH4 and SH3, predicted functions of proteins, and best matches with database**.

					**Predicted protein**						**Aligned protein**		
**ORF**	**SH4 ORF,%**	**SH3 ORF, %**	**Start**	**Stop**	**Size(aa)**	**MW(kDa)**	**pI**	**SD sequence (TAAGGAGGT) (5′-3′)^a^**	**Predicted function**	**Blast (extent; %aa identity^b^**	***E* Value**	**Size (aa)**	**Accession number**
1	ORF1, 100		934	1137	68	7.9	6.1	ATAGGATAAacaag**ATG**		metaG-MbCM1_078 [*Synechococcus* phage metaG-MbCM1] (28/59; 47%)	3.00E-11	59	YP_007001569.1
2	ORF2, 100		1134	1658	175	20.2	5.7	TAAGGAACTacaatc**ATG**		CPT_Seurat66 [*Escherichia* phage Seurat](87/167; 52%)	6.00E-48	167	YP_009152010.1
3	ORF3, 100	ORF3, 84	1652	1807	52	5.9	9.4	AGGTGAGGTcatcaag**ATG**		gp0.35 [*Enterobacteria* phage EcoDS1] (47/50; 94%)	2.00E-27	50	YP_002003737.1
4	ORF4, 100		2006	2173	56	5.9	8.3	ATAGGAGTTaact**ATG**		PE3_004 [*Escherichia* phage PE3-1] (48/55; 87%)	2.00E-24	55	YP_009044252.1
5	ORF5, 100	ORF5, 99	2177	2374	66	7.5	11.0	GCGGGATAAacc**ATG**		gp0.6 [*Enterobacteria* phage EcoDS1](64/65; 98%)	4.00E-37	65	YP_002003739.1
6	ORF6, 100	ORF6, 56	2374	2700	108	12.1	9.3	TTGGGAGCAaactgta**ATG**		PE3_006 [*Escherichia* phage PE3-1] (76/108; 70%)	8.00E-48	130	YP_009044254.1
7	ORF7, 100	ORF7, 93	2798	5479	894	100.5	7.6	CAAGGACTTtaagt**ATG**	RNA polymerase	gp1 [*Cronobacter* phage Dev2] (883/893; 99%)	0.0	893	YP_009005115.1
8	ORF8, 100	ORF8, 78	5492	5692	67	7.3	9.7	TAAGGAGGCatctac**ATG**		gp1.1 [*Cronobacter* phage Dev2] (66/66; 100%)	6.00E-38	66	YP_009005116.1
9	ORF9, 100		5771	6250	160	18.6	9.3	AGAGGTTGAcact**ATG**		gp1.06 [*Cronobacter* phage Dev2](155/159; 97%)	1.00E-110	159	YP_009005117.1
10	ORF10, 100	ORF10, 98	6339	6518	60	6.8	10.2	ACTGGAGATttaacc**ATG**		gp1.15 [*Cronobacter* phage Dev2](58/59; 98%)	4.00E-33	59	YP_009005118.1
11	ORF11, 100	ORF11, 22	6522	6809	96	11.2	6.3	GTAGGAGCGtaagac**ATG**		PE3_010 [*Escherichia* phage PE3-1](81/95; 85%)	5.00E-56	95	YP_009044258.1
12	ORF12, 99	ORF12, 75	6827	7897	357	40.2	5.5	TCTGGAGACattaacg**ATG**	DNA ligase	gp1.3 [*Enterobacteria* phage EcoDS1] (318/357; 89%)	0.0	365	YP_002003747.1
13	ORF13, 100	ORF13, 49	8019	8273	85	9.85	9.9	AGAGGAGAAacctt**ATG**		gp1.6 [*Enterobacteria* phage EcoDS1] (73/84; 87%)	6.00E-47	84	YP_002003748.1
14	ORF14, 100	ORF14, 67	8273	8593	107	12.2	6.9	CAAGGAGGAgttcta**ATG**		gp1.7 [*Cronobacter* phage Dev2] (85/107; 79%)	5.00E-50	116	YP_009005122.1
15	ORF15, 100	ORF16, 65	8672	8887	72	8.2	4.5	GAAGGAGAAaggact**ATG**	Bacterial RNA polymerase inhibitor	gp2 [*Cronobacter* phage Dev2] (48/54; 89%)	3.00E-25	54	YP_009005123.1
16	ORF16, 100	ORF17, 85	8935	9633	233	25.4	4.8	CTAGGAGATttacaccg**ATG**	Helix-destabilizing protein	gp2.5 [*Cronobacter* phage Dev2] (229/232; 99%)	2.00E-164	232	YP_009005124.1
17	ORF17, 100	ORF18, 43	9870	10088	72	8.3	9.9	TAAGAAGCAt**ATG**	Endonuclease	gp3 [*Cronobacter* phage Dev2] (72/72; 100%)	2.00E-44	139	YP_009005125.1
18	ORF18, 100	ORF19, 81	10085	10309	75	8.6	9.7	AAAGGAGCTaagaa**ATG**		gp3.2 [*Cronobacter* phage Dev2] (74/74; 100%)	3.00E-45	74	YP_009005126.1
19	ORF19, 98	ORF20, 90	10299	10757	153	16.9	8.8	GCTGGTGGTgtaca**ATG**	N-acetylmuramoyl-l-alanine amidase	gp3.5 [*Cronobacter* phage Dev2] (152/152; 100%)	8.00E-109	152	YP_009005127.1
20	ORF20, 100	ORF21, 61	10772	10984	71	7.4	10.1	CAAGGAGTAttaac**ATG**		gp3.7 [*Cronobacter* phage Dev2] (69/70; 99%)	1.00E-28	70	YP_009005128.1
21			11142	11507	122	13.9	10.1	GCGGGATAAacc**ATG**	HNH endonuclease	gp3.8 [*Enterobacteria* phage T7] (65/121; 54%)	1.00E-40	121	NP_041974.1
22	ORF21, 97	ORF22, 90	11482	13179	566	62.2	5.1	TAAGGAGGCtc**ATG**	Primase/Helicase	gp4 [*Cronobacter* phage Dev2] (545/566; 96%)	0.0	567	YP_009005129.1
22B	ORF21B, 98	ORF22B, 92	11782	13179	465	51.3	5.2	TTGGGTAGGc**ATG**	Primase/Helicase	gp4 [Cronobacter phage Dev2] (475/465; 98%)	0.0	567	YP_009005129.1
22.2	ORF21.2, 61	ORF22.2, 62	12908	13255	115	13.0	9.0	AAAGGTAAGtctc**ATG**		gp4.2 [Enterobacteria phage K1F] (63/107; 56%)	2.00E-25	107	CAJ29367.1
23	ORF22, 66	ORF23, 69	13182	13766	194	21.2	4.7	CAACGACTTctgacc**ATG**		gp4.1 [*Cronobacter* phage Dev2] (168/177; 95%)	1.00E-117	177	YP_009005130.1
24	ORF23, 99	ORF24, 90	13837	16008	724	80.9	7.0	ATAGGAGACatt**ATG**	DNA polymerase	gp5 [*Cronobacter* phage Dev2] (717/723; 99%)	0.0	723	YP_009005131.1
25	ORF24, 97	ORF26, 96	16008	16292	95	10.5	5.2	GAAGGAGTGtcacta**ATG**	HNS binding protein	gp5.5 [*Cronobacter* phage Dev2] (92/94; 98%)	4.00E-58	94	YP_009005133.1
26	ORF25, 100	ORF27, 100	16289	16498	70	7.4	9.0	ATTCGAGGTcaaacg**ATG**		gp21 [*Enterobacteria* phage K1F] (69/69; 100%)	5.00E-43	69	YP_338112.1
27	ORF26, 100		16495	16770	92	9.9	5.3	GGAGGCTGTct**ATG**		ASC_0027 [*Klebsiella* phage K11](37/68; 54%)	2.00E-19	68	YP_002003815.1
28	ORF27, 99	ORF28, 90	16763	17629	289	32.9	5.4	AAAGGAGGTctgcggg**ATG**	Exonuclease	gp6 [*Cronobacter* phage Dev2] (284/288; 99%)	0.0	288	YP_009005135.1
29	ORF28, 100	ORF29, 56	17837	18109	91	9.9	5.2	AGAGGAGACtttaag**ATG**		gp6.5 [*Cronobacter* phage Dev2] (90/90; 100%)	8.00E-58	90	YP_009005136.1
30	ORF29, 100	ORF30, 92	18120	18344	75	7.6	6.2	AAAGGAGGGact**ATG**	Head protein	gp6.7 [*Cronobacter* phage Dev2] (74/74; 100%)	1.00E-42	74	YP_009005137.1
31	ORF30, 100	ORF31, 91	18348	18752	135	15.5	6.1	ACATGGGGTAAGac**ATG**		gp34 [*Citrobacter* phage CR44b] (127/135; 94%)	7.00E-89	185	YP_009007168.1
32	ORF31, 100	ORF32, 96	19011	20579	523	57.2	4.6	GCAGGAGGTgacaa**ATG**	Head to tail connector protein	gp8 [*Cronobacter* phage Dev2] (522/522; 100%)	0.0	522	YP_009005140.1
33	ORF32, 99	ORF33, 81	20684	21565	294	31.7	4.4	AAAGGAGAAcgactca**ATG**	Capsid assembly protein	gp9 [*Cronobacter* phage Dev2] (290/293; 99%)	0.0	293	YP_009005141.1
34	ORF33, 99	ORF34, 94	21697	22746	350	36.4	5.8	ATAGGAGAAttatcat**ATG**	Major capsid protein	gp10 [*Cronobacter* phage Dev2] (347/349; 99%)	0.0	349	YP_009005142.1
35	ORF34, 100	ORF35, 97	23067	23633	189	21.3	4.4	TAAGGAGGGcct**ATG**	Tail tube protein A	gp11 [*Cronobacter* phage Dev2] (187/188; 99%)	3.00E-135	188	YP_009005144.1
36	ORF35, 99	ORF36, 87	23645	26014	790	87.6	5.8	ATAGGAGGTgat**ATG**	Tail tube protein B	gp12 [*Cronobacter* phage Dev2] (779/789; 99%)	0.0	789	YP_009005145.1
37	ORF36, 97	ORF37, 83	26090	26548	153	17.5	6.9	ATAGGAGACttt**ATG**	Internal virion protein A	gp13 [*Cronobacter* phage Dev2] (150/152; 99%)	6.00E-107	152	YP_009005146.1
38	ORF37, 98	ORF38, 91	26669	27256	196	20.4	6.8	CCGGGAGGTgaaag**ATG**	Internal virion protein B	gp14 [*Cronobacter* phage Dev2] (194/195; 99%)	1.00E-136	195	YP_009005147.1
39	ORF38, 99	ORF39, 89	27268	29550	761	85.3	5.5	ATAGGAGGAcca**ATG**	Internal virion protein C	gp15 [*Cronobacter* phage Dev2] (749/760; 99%)	0.0	760	YP_009005148.1
40	ORF39, 99	ORF40, 92	29556	33452	1299	141.0	5.8	TAAGGAGTAataaca**ATG**	Internal virion protein D	gp16 [*Cronobacter* phage Dev2] (1282/1298; 99%)	0.0	1298	YP_009005149.1
41	ORF40, 100	ORF41, 79	33520	36018	832	91.3	6.4	TAAGGAGGCcca**ATG**	Tail fibers	gp17 [*Cronobacter* phage Dev2] (805/832; 97%)	0.0	832	YP_009005150.1
42	ORF41, 100	ORF42, 95	36065	36259	65	6.9	8.0	AACGGAGGTatt**ATG**	Lysis protein	gp17.5 [*Cronobacter* phage Dev2] (64/64; 100%)	4.00E-37	64	YP_009005151.1
43	ORF42, 100	ORF43, 97	36256	36519	87	10.1	4.8	AGTGGAGGTaagac**ATG**	DNA packaging protein	gp18 [*Cronobacter* phage Dev2] (87/87; 100%)	1.00E-54	87	YP_009005152.1
44	ORF43, 100	ORF44, 73	36624	37073	150	16.9	8.8	CGAGGAGGGcaact**ATG**	Endopeptidase Rz	gp18.5 [*Cronobacter* phage Dev2] (147/149; 99%)	3.00E-101	149	YP_009005153.1
44.7	ORF43.7, 100	ORF44.7, 72	36727	36999	90	9.8	9.6	GAAGGTAAGca**ATG**	Endopeptidase Rz1	gp18.7 [Enterobacteria phage EcoDS1] (60/89; 67%)	2.00E-31	91	YP_002003785.1
45	ORF44, 100	ORF45, 94	37103	38833	577	65.1	5.3	TCAGGCGCTt**ATG**	Maturation protein	gp19 [*Cronobacter* phage Dev2] (574/577; 99%)	0.0	587	YP_009005154.1
45.2	ORF44.2, 100		37752	37877	41	4.8	12.1	TATCCTCGTg**ATG**		gp19.2 [*Enterobacteria* phage K1F] (27/55; 49%)	7.00E-09	55	CAJ29396.1
46	ORF45, 100	ORF46, 94	39124	39282	53	5.5	9.3	GTATGTAGC**ATG**		gp19.5 [*Cronobacter* phage Dev2] (52/52; 100%)	2.00E-27	52	YP_009005155.1

a Start codon indicated in boldface; Match to SD sequence is indicated by underlining; SD position is indicated in uppercase.

b ^b^The number of identical amino acids/The total number of amino acids of smallest protein.

The genomes of the five isolated phages are co-linear and share the same genomic organization as phage T7 with what seems to be early-, middle-, and late-expressed regions. The early genes are usually involved in host takeover and conversion of the host metabolism for the benefit of phage production (Pajunen et al., 2001). This region is also characterized by the presence of an RNA polymerase responsible for the transcription of all the middle- and late-expressed genes. The middle-expressed region includes genes responsible for DNA metabolism while the late region contains genes coding for structural proteins.

### Proteomic analyses

The structural proteome of one phage representing each of the three subgroups (phage SH1 for group 1, SH3 for group 2A and SH4 for group 2B) was analyzed. Purified phages were analyzed by LC-MS/MS and the results are presented in Table 6. For phage SH1, 11 proteins were detected with an amino acid coverage ranging from 12 to 65%. Ten of the 11 genes coding for these proteins were located in the presumably late-expressed module, as expected for genes coding for structural proteins. The other protein (ORF19) was a N-acetylmuramoyl-L-alanine amidase probably involved in host lysis and it had the lowest coverage (12%). Its gene was located in the middle-expressed region. It is unclear if this protein is in the phage structure or if it is a non-structural phage protein that was carried over from the phage purification process.

**Table 6:** **Identified peptides for phages SH1, SH3 and SH4 and their predicted functions**.

**Phage**	**Start**	**Stop**	**ORF**	**Predicted function**	**Mass (kDa)**	**Exclusive unique peptide**	**Coverage (%)**
SH1	11614	12069	19	N-acetylmuramoyl-L-alanine amidase	17	2	12
	18713	19039	31	Capsid protein	12	2	36
	19067	19387	32	Host specficity protein B	11	3	29
	19398	21005	33	Capsid to tail joining protein	59	29	65
	22196	23236	35	Major capsid protein	37	22	60
	23426	24016	36	Tail tubular protein A	22	5	23
	24032	26437	37	Tail tubular protein B	90	32	44
	26923	27516	39	Internal virion protein B	21	12	65
	27519	29762	40	Internal virion protein C	85	36	59
	29781	33743	41	Internal virion protein D	144	63	57
	33815	35791	42	Tail fibers protein	70	21	48
SH3	18963	20531	32	Capsid to tail connector protein	57	26	67
	20676	21560	33	Capsid assembly protein	32	7	21
	21687	22730	34	Major capsid protein	36	8	50
	22925	23491	35	Tail tube protein A	21	5	32
	23503	25872	36	Tail tube protein B	88	24	37
	26404	26991	38	Internal virion protein B	20	10	65
	27003	29285	39	Internal virion protein C	85	25	42
	29290	33177	40	Internal virion protein D	141	45	45
	33243	35747	41	Tail fibers protein	91	20	33
SH4	18577	20145	31	Capsid to tail connector protein	57	5	39
	21263	22312	33	Major capsid protein	36	15	40
	23211	25580	35	Tail tube protein B	88	10	24
	26111	26698	37	Internal virion protein B	20	2	32
	26710	28992	38	Internal virion protein C	85	14	29
	28998	32894	39	Internal virion protein D	141	14	20
	32962	35460	40	Tail fibers protein	91	9	18

For phage SH3, 9 structural proteins were detected with coverage ranging from 21 to 67%, while for phage SH4, 7 structural proteins were identified with coverage ranging from 18 to 40%. For these two phages, all the proteins detected were structural proteins from the capsid, head-tail joining, tail, tail tube, and tail fibers.

### DNA polymerase phylogeny

**Figure 6:**
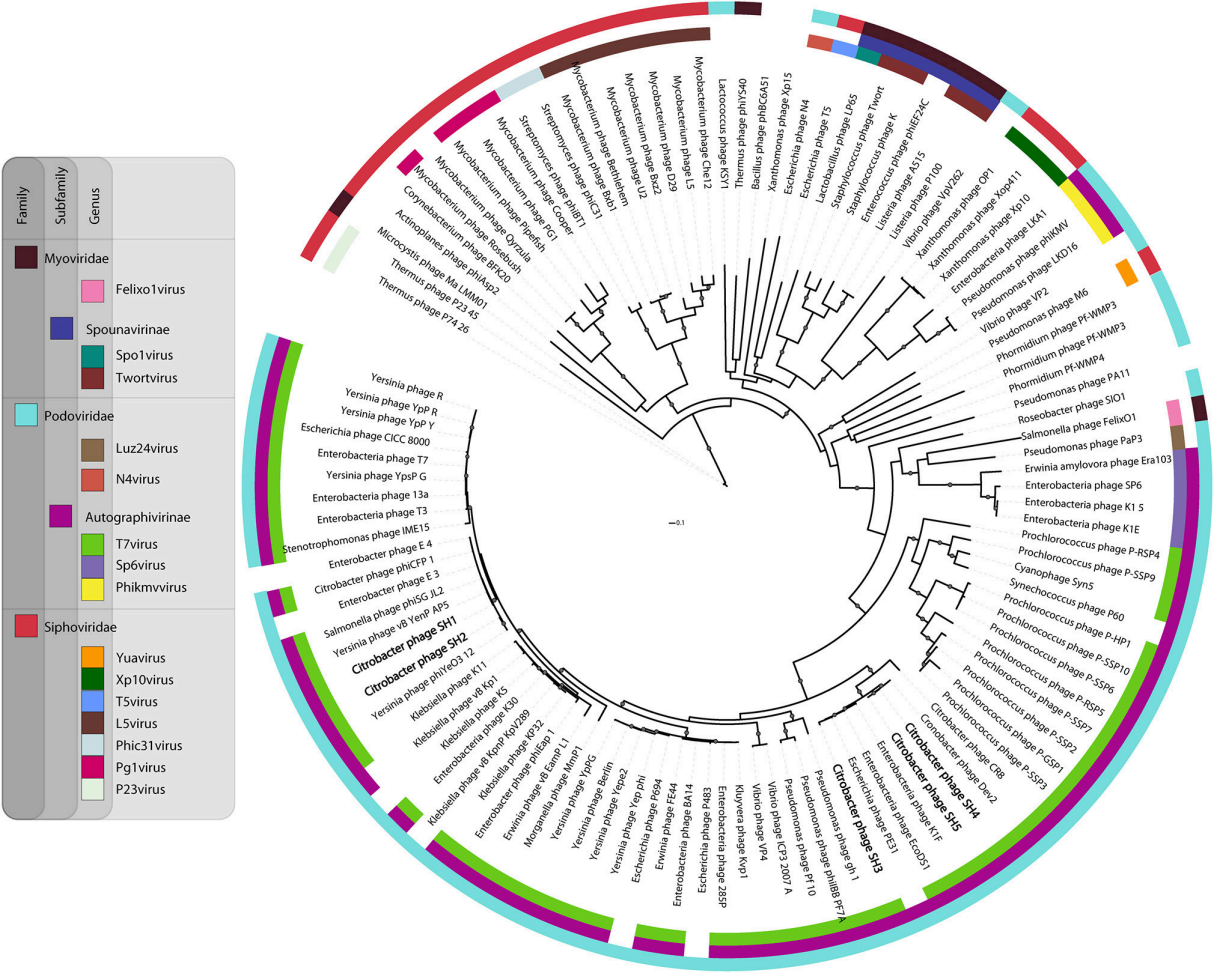
**Phylogenetic relationship between selected phage DNA polymerase sequences**.

Because the five *Citrobacter* podophages belong to the *T7virus* genus, we compared in greater detail their relationships with other characterized similar phages available in public database (Figure 6). The T7 DNA polymerase is a conserved protein often used to study the global distribution and diversity of podophages, in a manner analogous to the 16S rRNA in bacteria (Breitbart et al., 2004). Based on DNA polymerase phylogeny, the five phages were confirmed to belong to the *T7virus* genus in the subfamily *Autographivirinae*. However, they mapped at two different sub-branches. Phages SH1 and SH2 were similar to *Yersinia* phages phiYeO3-12 and vBYenP AP5, *Salmonella* phage phiSG-JL2, *Citrobacter* phage phiCFP-1, and *Enterobacter* phages E3 and E4. They were also closer to the prototype phage T7 than the other three phages characterized here. Phages SH3, SH4, and SH5 were part of the same clade of t7viruses as SH1 and SH2, but clustered in different subgroups. Phage SH3 was related to *Enterobacteria* phages K1F and EcoDS1, and *Escherichia* phage PE3-1. Phages SH4 and SH5 were more related to *Cronobacter* phage Dev2. Taken altogether, despite the differences between these two groupings, SH1/SH2 and SH3/SH4/SH5 seem to be derived from a common ancestor.

### Comparison between phages SH1/SH2, phiYeO3-12, and coliphage T7 (Group 1)

Pairwise analyses between the deduced proteomes of phages SH1 and SH2 revealed 31 proteins (out of 53) with more than 95% identity (Table 4). Among them, seven (ORF8, ORF18, ORF23, ORF26, ORF29, ORF39, ORF44) were 100% identical, including two proteins with predicted functions, ORF39/internal virion protein B and ORF44/DNA packaging protein A. Phage SH1 also shared more than 95% identity with 31 proteins of *Yersinia* phage phiYeO3-12 including seven proteins with 100% identity (ORF8, ORF20, ORF22, ORF23, ORF25, ORF26, ORF29). Phage SH2 shared more than 95% identity with 34 proteins of *Yersinia* phage phiYeO3-12 including 13 proteins with 100% identity (Table 4). Phage SH2 seems more related to phage phiYeO3-12 than phage SH1. One of the most notable differences between phages SH1 and SH2/phiYeO3-12 was mobile elements. Phage SH1 is missing the homing endonuclease encoded on phages SH2 and phiYeO3-12 (ORF23^SH2^/ORF5.3 ^phiYeO3-12^). However, phage SH1 has another homing endonuclease (ORF17), which is absent in both genomes of SH2 and phiYeO3-12. ORF17^SH1^ is homologous to a homing endonuclease found on *C. rodentium* phage CR44b (46%). Phage SH1 is also missing ORF35^SH2^/ORF10B^phiYeO3-12^ a minor capsid protein (Condron et al., 1991). On the other hand, the tail fiber protein of phage phiYeO3-12 shares only 68 and 67% identity with the tail fiber proteins of SH2 and SH1, respectively, which could explain the divergent host ranges between SH1/SH2 and phiYeO3-12. In fact, phages SH1 and SH2 were not able to infect the host strain *Yersinia enterocolitica* 6471/76-c (HER1249) of phage phiYeO3-12.

In addition, phages SH1 and SH2 shared 11 proteins with more than 80% amino acid identity with coliphage T7, including the RNA polymerase (ORF1^T7^ and ORF6^SH1/SH2^). The T7 RNA polymerase initiates transcription by exclusively recognizing its own promoters to ensure fast and efficient transcription of phage DNA. It is also involved in DNA replication, maturation and packaging (Studier and Moffatt, 1986; Zhang and Studier, 2004).

Another T7 protein homologous to SH1/SH2 proteins was ORF2.5^T7^ (homologous to ORF16^SH1^ and ORF15^SH2^), which is a single-stranded DNA binding protein. The *orf2.5*^*T*7^ gene is essential for phage DNA replication and recombination (Scaltriti et al., 2009, 2013). The N-acetylmuramoyl-L-alanine amidase ORF3.5^T7^ was also related to ORF19^SH1^ and ORF17^SH2^. This lysozyme is involved in cell lysis but may also inhibit transcription by binding to the RNA polymerase to ensure a controlled burst of late transcription (Inouye et al., 1973; Moffatt and Studier, 1987). ORF21^SH1^ and ORF19^SH2^ were similar to the T7 primase/helicase, ORF4^T7^. This primase/helicase activity is essential for DNA replication (Rosenberg et al., 1992) as the helicase catalyzes strand displacement during DNA replication while the primase is involved in the synthesis of the DNA lagging-strand (Mendelman et al., 1992).

The ORF5.7 protein of phage T7 shared a high level of identity with ORF26^SH1^ and ORF25^SH2^. ORF5.7 stimulates the expression of gene 5.5 which encodes a H-NS binding protein (Zhu et al., 2012). When gene 5.5 is missing, the phage plaque and the burst sizes are reduced (Owen-Hughes et al., 1992; Liu and Richardson, 1993). The H-NS binding protein inhibits the function of the highly conserved host histone-like nucleoid structuring (H-NS) protein, which influences gene expression, recombination and transcription.

A notable difference between phage T7 and phages SH1/SH2 was in their antirestriction proteins (gp0.3^T7^/ORF1^SH1/SH2^). Restriction-modification (R-M) systems are well-known resistance mechanisms used by bacteria to block phage replication (Labrie et al., 2010). Phages also have several means to bypass these systems (Samson et al., 2013). The Phage T7Ocr (overcoming classical restriction, ORF0.3) protein mimics the DNA phosphate backbone, interacting directly with the type R-M*Eco*KI enzyme, and interfering with the activity of this system (Atanasiu et al., 2002; Stephanou et al., 2009). At the same genomic location (Figure 5), the phage SH1 and SH2 *orf1* genes code for a putative S-adenosyl-l-methionine hydrolase, homologous to gp0.3^phiYeO3-12^, which destroys *S*-adenosyl-l-methionine, an essential R–M cofactor (Studier and Movva, 1976). The Ocr protein of phage T7 does not have the hydrolase activity. However, the Ocr protein of *E. coli* podophage T3, whose gene is located at the same genomic position, possesses this hydrolase activity.

### Comparison between phages SH3 and K1F (Group 2A)

The deduced proteome of phage SH3 (49 ORFs) ranged from 30 to 75% identity to the proteins of phages SH1 and SH2. However, phage SH3 had eight proteins with more than 95% identity to proteins of phages SH4 and SH5, including 100% identity between ORF27^SH3^ and ORF25^SH4^/ORF26^SH5^ (Table 5). Otherwise, the closest phage to SH3 was coliphage K1F with 23 proteins sharing more than 95% identity. Of these, four proteins are 100% identical, including two with a known function (lysis protein and DNA packaging protein). Genetic differences were noted between *Citrobacter* phage SH3 and *E. coli* phage K1F and the most important difference lies in tail fibers (Gp17^K1F^/ORF41^SH3^) that consist of two domains. The N-terminal domain is responsible for attachment to the phage tail and the C-terminal domain is involved in the recognition of and adsorption to the host LPS (Kajsík et al., 2014). The N-terminal parts of the tail fibers of both K1F and SH3 shared a region with the phage T7 tail fiber. However, the central catalytic portion of Gp17^K1F^ encodes an endosialidase to penetrate the host polysaccharide capsule (Scholl and Merril, 2005) while ORF41^SH3^ contains a domain of the SGNH hydrolase superfamily like the tail fibers of phages Dev2, SH4, and SH5. However, the C-terminal part of ORF41^SH3^ is different than the tail fibers of phages SH4, SH5, and Dev2, which explains its different host range. The SH3 genome is also missing the putative group I intron present within the DNA polymerase of K1F (gp5.3) which encodes a homing endonuclease.

### Comparison between phages SH4/SH5 and Dev2 (Group 2B)

Of the 45 genes of phage SH5, 33 were 100% identical to genes of phage SH4. Ten of these genes are also 100% identical to the *T7virus Cronobacter* phage Dev2 genes. These conserved genes suggest that the three phages may be derived from a common ancestor. In addition, phages SH4 and SH5 have more than 95% aa identity with almost all of the phage Dev2 structural proteins. Interestingly, the putative tail fiber proteins ORF40^SH4^ and ORF41^SH5^ were 99% identical to tail fiber gp17 of phage Dev2, suggesting a similar host range. We received phage Dev2 and tested its host range in parallel with phages SH4 and SH5 on the 31 bacterial strains available. The three phages were able to lyse the same strains, *C. freundii* CF3, *C. freundii* CF4, and *C. turicensis* 290708/07.

Phages SH4 and SH5 are missing the genes coding for gp5.1- and gp10.1-like located in the late-expressed region, found in Dev2 (Kajsík et al., 2014). Most genomic differences between SH4/SH5 and Dev2 were located in the early-expressed region. ORF21 of phage SH5, which encodes an HNH endonuclease with a zinc-binding motif involved in different steps of phage development (Anba et al., 2002), was missing from phages SH4 and Dev2. However, ORF21 shares 54% identity with T7 gp3.8.

The SH4 and SH5 proteins with the lowest similarity were ORF22^SH4^ (132 aa) and ORF23^SH5^ (194 aa) but these were still 66% identical. Their amino acid sequences could be aligned perfectly at the C-terminal end but ORF22^SH4^ is missing the N-terminal portion of ORF23^SH5^. A mutation may have occurred as we noticed the lack of a T base at the ATG codon of ORF22^SH4^. ORF23^SH5^ had 95% identity to gp4.1 of phage Dev2 but its function is unknown.

## Discussion

In this study, we isolated and characterized five virulent *Podoviridae* phages infecting *C. freundii*, an emerging pathogenic bacterial species (Samonis et al., 2008). Genome analyses showed that the five newly isolated phages belong to the *Autographivirinae* subfamily and the *T7virus* genus. Their morphological and genomic properties allowed us to separate them into two different groups, group 1 (phages SH1 and SH2) and group 2 (phages SH3, SH4, and SH5). However, the two groups are co-linear and share conserved genomic organization. They are flanked by terminal repeats involved in concatemer formation, DNA packaging, and particle maturation (Chung et al., 1990). Despite their small size (close to 40 kb), the five phage genomes encode the usual modules with genes coding for proteins involved in DNA replication, transcription regulation, morphological proteins, lysis proteins, as well as DNA maturation and packaging. As such, they have very compact genomes with overlapping genes (Mendelman et al., 1992) as more than 90% of the five genome sequences were predicted to encode proteins. For phages SH1, SH3, and SH4 almost all the predicted structural proteins were detected by LC-MS/MS, showing that they are indeed transcribed and translated.

Another reason for sequencing the new phage genomes is to provide a clearer view about the dynamics of phage populations over space and time. Based on genomic and proteomic identification, we could define evolutionary relationships between these podophages (Brüssow and Hendrix, 2002). For example, phage T7 was isolated in 1945 (Delbrück, 1945), phage phiYeO3-12 from sewage in 1988 in Finland (Al-Hendy et al., 1991), phage K1F from sewage in 1984 in the USA (Scholl and Merril, 2005), and phage Dev2 was recently isolated from sewage in Slovakia (Kajsík et al., 2014). All five *C. freundii* phages characterized in this study were isolated from different sewage samples collected in Tunisia in 2014. These phages are geographically and temporally distant but from an evolutionary perspective, these phages likely shared a common ancestor.

As phages tend to coevolve with their bacterial hosts (Skurnik and Strauch, 2006) and *C. freundii* can produce enterotoxins (Guarino et al., 1987), we inspected the five phage genomes for the presence of host related genes, particularly those coding for known virulence-factors or integrase. No such genes were found, indicating that they are truly lytic phages as well as suggesting that they may be safe for therapeutic or prevention applications. Moreover, it was relatively easy to purify them and we obtained highly concentrated phage preparations. Conversely, these phages were inactivated at very acidic pH (2–3), suggesting that they may not survive in high numbers after passage through the gastrointestinal tract or in highly acidic foods. Others have shown that microencapsulation in alginate-chitosan microspheres significantly improved the survival and stability of phages under harsh acidic conditions (Ma et al., 2008). Finally, their limited host range suggests that they should be used in combination to maximize strain coverage. Of note, no CRISPR-Cas systems were found in the *C. freundii* genomes analyzed.

Taken altogether, the newly characterized *Podoviridae* phages SH1, SH2, SH3, SH4, and SH5 have appealing properties for prophylactic or therapeutic use to control the proliferation of *C. freundii* infections. The analyses of these *Citrobacter* phages also provided new evolutionary relationships with the expanding group of phages belonging to the *T7virus* genus, including with phages infecting *Cronobacter* and *Yersinia* species of the *Enterobacteriaceae* family.

## Author contributions

SM, KS, RK conceived and designed the study and afforded the materials. SH performed the experiments, analyzed the data and drafted the manuscript. GR participated in the data analysis and helped in the coordination of the experiments. DT did the sequencing and the electron microscopy. SL designed the figures and helped in the bioinformatics analysis. SM critically revised the manuscript. All authors read and approved the manuscript.

### Conflict of interest statement

The authors declare that the research was conducted in the absence of any commercial or financial relationships that could be construed as a potential conflict of interest.
